# Gut-Associated Lymphoid Tissue: A Key Tissue Inside the Mucosal Immune System of Hens Immunized with *Escherichia coli* F_4_

**DOI:** 10.3389/fimmu.2017.00568

**Published:** 2017-05-22

**Authors:** Maria F. Peralta, Alejandra Magnoli, Fabrisio Alustiza, Armando Nilson, Raúl Miazzo, Adriana Vivas

**Affiliations:** ^1^Produccion Animal, Facultad de Agronomía y Veterinaria, Universidad Nacional de Rio Cuarto, Córdoba, Argentina; ^2^Anatomia Animal, Facultad de Agronomía y Veterinaria, Universidad Nacional de Rio Cuarto, Córdoba, Argentina

**Keywords:** gut-associated lymphatic tissue, B lymphocytes, hen, *Escherichia coli* F_4_, immunoglobulin A, immunoglobulin Y technology, flow cytometry, histomorphometry

## Abstract

Immunoglobulin Y (IgY) is the predominant antibody found in hen’s (*Gallus domesticus*) egg yolk. This antibody, developed against several microorganisms in hen egg yolk, has been successfully used as an alternative to immunoglobulins from mammals for use in immunodiagnostics and immunotherapy. Enteropathogenic *Escherichia coli* (E.coli) F_4_ is the main etiological agent associated with swine neonatal diarrhea, and it causes notable economic losses in swine production. The aim of the present study was to evaluate the relationship between humoral immune response and the activation of gut-associated lymphoid tissue (GALT) in laying hens intramuscularly immunized with *E. coli* F_4_. Adult laying Shaver hens were immunized with a bacterin based on an inactivated lysate *E. coli* F_4_ strain that was originally isolated from neonatal piglet diarrhea, following a recommended schedule. The percentage of B lymphocytes in blood and spleen homogenates was determined by flow cytometry. Villi histomorphometry and the size of germinal centers (GC) activated in GALT and the spleen were measured in histological samples either stained with hematoxylin/eosin or through immunofluorescence. Antibody and isotype-specific antibodies in serum and egg yolk were measured using indirect enzyme-linked immunosorbent assay (ELISA). Secretory and serum immunoglobulin A (IgA) were measured by ELISA tests. Laying hen with intramuscular immunization with *E. coli* F_4_ lysate, activated both mucosal and systemic protection. Mucosal protection was provided through B lymphocytes, and most of them were activated on Peyer’s patches and esophageal tonsils, in GALT. Furthermore, increased B lymphocyte number in the *lamina propria* of the gut, and increased intraepithelial plasmatic cell number, produced high levels of mucosal IgA. Activated B lymphocytes interacted with absorptive cells, immune cells, and microbiota in the gut, producing signals that were translated into a powerful physical defense by producing a greater volume of mucin from an increased number of goblet cells. Systemic protection was provided through B lymphocyte activation of spleen GC, which produced hugely specific IgY serum levels. One week later, this specific IgY was deposited in the yolk. This suggests that GALT is a key immunologic tissue inside the mucosal immune system, acting as the “command center” for humoral reaction.

## Introduction

Over recent years, there has been an increase in the use of oral passive immunotherapy. One novel protective strategy to achieve public health in humans and domestic animals is the production of specific immunoglobulin Y (IgY) in laying hens. This IgY is produced after the immunization of laying hens, when the humoral immune response (IR) is activated and produces specific IgY in the blood. After approximately a week, this IgY is transported to the yolk to confer natural passive immunity to the embryo and offspring (1, 2). This specific IgY can be easily purified and applied to medical fields and research, thus replacing antibiotic therapy by immunotherapy. This is known as *IgY biotechnology* (1–3) and has been shown to be effective in the inhibition of several enteric pathogens including *Escherichia coli* (*E. coli*) in pigs (3–6) and neonatal calves (3). Consequently, antibodies (Ab) developed against, for example, *E. coli* F_4_ could be suitable alternatives for use in newborn piglets, to diminish or eliminate fatal diarrhea infections (4).

Different research results have been reported concerning IgY levels, both in total and those specifically present in the serum or yolk, after laying hens have been immunized with different enteric pathogens employed as antigens (Ags) (5, 7–9). However, there is limited information about immune mechanisms involved in avian IR, such as the activation of secondary lymphoid organs [for example, the mucosal immune system (MIS)] and the spleen after immunization. Furthermore, avian immune systems differ from those of mammals (for example, in Toll-like receptor, chicken growth factor, and chemokine/chemokine receptor repertoires), and the lymph nodes are absent (2). Moreover, in poultry, MIS is the main inductor site for IR when the primary lymphoid organs involute at 20 weeks of age. However, the interrelated mechanisms within the humoral IR that lead to the production of IgY have not yet been studied. A well-developed MIS is crucial for hens, because it is the first immunological defensive barrier against oral and airborne pathogens (2). MIS comprises mucosae-associated lymphoid tissue (MALT) (inductor site), where the specific IR begins, and the *lamina propria*, as well as effector sites for Ab production and immune cell-mediated responses (10). MALT includes the Harderian glands, bronchial-associated lymphoid tissue, nasopharyngeal lymphoid tissue, and gut-associated lymphoid tissue (GALT) (2). There is a constant migration of Ag-primed immune cells from inductive to effector sites. This phenomenon constitutes the cellular basis for the common MIS (2, 10, 11).

In poultry, GALT encompasses esophageal tonsils, pyloric tonsils, Meckel’s diverticulum, Peyer’s patches, and two cecal tonsils. Cecal tonsils are functionally and anatomically the most important organs of the GALT (2). Gut mucosa is exposed to the microbiota and commensal bacteria that are required for the processing of nutrients and the education of the local immune system shortly after birth (11, 12). The interplay between microbiota, intestinal epithelium, and the innate and adaptive immune cells is crucial for understanding homeostasis, as it improves the dominance of regulatory networks that prevent inflammation or immune-mediated diseases. A comprehensive understanding of these interactions will provide tools that can maximize performance and health in poultry, which are commonly raised at high population densities (10, 11, 13, 14).

Although there are studies regarding the immunization schedule for IgY production (i.e., different methods of purification and quantification from eggs), research on humoral immune mechanisms after laying hen immunization with enteric bacterin is limited. For example, the activation of GALT has not been studied.

Therefore, the aim of the present study was to evaluate the relationship between the humoral IR and the activation of GALT in laying hens intramuscularly immunized with *E. coli* F_4_ lysate.

## Materials and Methods

### Bacterial Strain and Immunogen

The immunogen was made using an enterotoxigenic *E. coli* (ETEC) (*E. coli* F_4_) strain with the following virulent factors: F_4_, LT, and STb, originally isolated from piglets with neonatal diarrhea on a farm in Córdoba Province, Argentina (4).

To extend the immunogen, ETEC was grown in Minca broth medium for 72 h at 37°C to overexpress fimbria Ags. After growth, the bacteria were disabled by incubating in 0.5% formaldehyde overnight. The disabled ETEC’s strain was pelleted by centrifugation (4,000 × *g* for 5 min), and the pellet was washed five times with sterile phosphate buffer saline (PBS). This was used as the immunogen (bacterin), a concentration of 10^9^ UFC/mL mixed with aluminum hydroxide 7% (employed as adjuvant) and stored at 4°C until needed for analyses (4).

### Animals

Forty-four Shaver hens (1-day-old) were obtained from a poultry farm (Monte Buey Avícola, Monte Buey, Córdoba, Argentina). The animals were kept in individual cages with food and water *ad libitum*, without contact with any other animals. At 19 weeks of age, the hens were divided into two groups: F_4_ (*n* = 25) and Control (*n* = 19).

The hens were fed a balanced ration for laying hens (Nutriarte, Córdoba-Argentina). A light/dark cycle of 16/8 h was used. Room temperature was 20 ± 2°C, and the relative humidity was between 55 and 60%.

All experiments were performed according to the institutional guidelines for animal care and experimentation. The protocol and procedures employed were reviewed and approved by the Ethics Committee of the Universidad Nacional de Rio Cuarto (15).

### Experimental Design

At 22 weeks of age, laying hens were immunized with 1 mL of bacterin (F_4_ group) or sterile PBS with 7% adjuvant (aluminum hydroxide; Control group); this was injected intramuscularly and distributed in both breast muscles. Booster immunizations were given at 2, 4, 6, and 8 weeks after the initial immunization. Eggs were manually collected every day from 23 to 42 weeks; egg yolks were separated from egg whites and stored at 4°C until needed for further analysis. Blood samples were taken from hens on the first day of immunization and every subsequent 15 days until the end of the test (57 days after the first immunization). Sera samples were preserved at −20°C for the determination of IgY and immunoglobulin A (IgA).

### Humoral IR Evaluation

#### Flow Cytometry

##### Blood Samples

Approximately 2 mL of blood was collected from the brachial wing vein of each hen using heparinized syringes and then transferred into heparinized tubes. Blood samples were processed with 1 h between each drawing. Mononuclear cells were separated from the whole blood by using density gradient separation with Histopaque-1077 (Sigma-Aldrich, St. Louis, USA) according to a previously established protocol (16). Briefly, 1.5 mL of blood was layered on 1.5 mL of Histopaque-1077 in a plastic tube. The samples were centrifuged at 400 × *g* for 30 min at room temperature. The resulting buffy coat of white cells above the erythrocytes was collected and transferred to a fresh tube. Cells were washed twice with 4 mL of PBS and centrifuged at 400 × *g* for 7 min. To eliminate erythrocytes that could interfere with B lymphocyte measurement. Final pellet was re-water suspended in PBS with 1% bovine serum albumin (Sigma-Aldrich). Cells were counted using a Neubauer counting chamber, always by the same person.

##### Spleen Samples

Immediately following death, half of the spleen of each laying hen was placed into a Petri dish with frost tamponed saline solution without Ca^2+^ and Mg^2+^ (CMF). The cells from the spleen were collected by mashing the tissue through a 60-µm nylon mesh into 5 mL of frost Roswell Park memory institute (RPMI)-1640. A further 2 mL of media was used to rinse any remaining cells through the mesh. The cells were separated by density gradient separation using Histopaque-1077 according to a previously established protocol (16, 17). Briefly, 1.5 mL RPMI-1640 with cells collected were layered on 1.5 mL of Histopaque-1077 in a plastic tube and centrifuged at 2,000 × *g* for 30 min; this was implemented a further two times. Viable lymphocyte counts were made using the trypan blue exclusion method, and the cells were diluted into a working concentration from 1 × 10^7^ cells/mL to a final volume of 2 mL of whole RPMI-1640.

##### Immunofluorescence Staining

The monoclonal anti-chicken antibody used in this research was Bu-1 a/b (AV20) coupled to fluorescein isothiocyanate (FITC; Santa Cruz Biotech). Bu-1 is expressed on avian B lymphocytes and used for single staining at 1:10 dilution of primary Ab (0.5 mg/mL) in PBS (pH 7.4). For blood samples, 50 µL of this dilution was mixed with 10^6^ B lymphocytes and kept at 4°C in the dark for 15 min. After this incubation period, cells were washed with 100 µL of PBS and centrifuged at 400 × *g* for 7 min. For spleen samples, 50 μL of the dilution were mixed with 10^7^ B lymphocyte spleen cells and kept at 4ºC in the dark for 20 min. After this incubation period, cells were washed with 100 μL of PBS and centrifuged at 400 × *g* for 7 min (16).

Fixation of blood and spleen B lymphocytes was performed according to a previously published procedure (18).

All samples underwent cytometry within 4 h of staining. Flow cytometry was performed on a BD FACS Canto II flow cytometer (San Jose, CA, USA). Green fluorescence (from FITC) was detected on the FL1 channel (530/30 nm), cells were analyzed at up to 10,000 events in the lymphocyte gate, based on forward and side scatters (19). Data were analyzed using FlowJo software (TreeStar, Inc.).

#### GALT and Spleen Histopathological and Immunofluorescence Evaluation

After necropsy, samples from esophageal, pyloric, and cecal tonsils and Peyer’s patches, spleen, and intestine (2 cm segment of the jejunal region before Meckel’s diverticulum) were fixed in buffered formalin (pH 7.4), dehydrated through graded alcohol to xylene, and paraffin embedded at 56–58°C for at least 3 h (1172601; Cicarelli). Subsequently, 3–4 µm sections were cut (Reichert-Jung ultramicrotome Leica RM 206) and placed on slides. Hematoxylin/eosin (H/E) staining was performed; sections were observed (Axiostar Plus Carl Zeiss microscope); and microphotographs were taken using a Canon PowerShot G6, 7.1 megapixels.

##### Immunofluorescence Evaluation

AV20-FITC was used to stain GALT and spleen sections of both the F_4_ and Control groups; these were used to measure germinal center (GC) that displays abundant fluorescent B lymphocytes using Image J software. Briefly, counting started from a point, moved through all the GC, and returned to the starting point. For this study, a digital camera (PowerShot G6, 7.1 megapixels, Canon Inc., Japan) was attached to a microscope (Axiophot, Carl Zeiss). In each spleen slide, 12 measurements of the GC were taken from each laying hen.

##### Histomorphometric Analysis

Hematoxylin–eosin stained intestinal sections from the F_4_, and Control groups were used to measure height, area, and perimeter of villi using a light microscope (Axiophot, Carl Zeiss). The data were processed using computer software (Axio Vision release). The height was the distance between the villous–crypt axis and the top of the villus. Villi area and perimeter were measured starting from the base thereof, crossing the edges with the cursor hairs and returning to the starting point. For each one of the variables, at least 15 measures of complete villi were taken from each slide of each hen.

#### Serum and Intestinal Mucosal Secretory IgA Determination

The total concentration of IgA was evaluated by IgA Enzyme-Linked Immunosorbent Assay (ELISA) kit (ab157691 ABCAM). Sera samples were tested according to the manufacturer’s instructions. To measure secretory IgA, 2-cm segments of the jejunal region near to Meckel’s diverticulum were excised and then exposed. Mucus was collected by scraping the mucosal surface of the intestine, which was then weighed to equal quantities. Thereafter, the mucus from each gut sample was suspended in a 4-fold volume of PBS (wt/wt), vortexed thoroughly, and centrifuged at 5,000 × *g* for 30 min at 4°C (51). After centrifugation, the supernatant was removed and stored at −80°C for further analysis. An ELISA test was performed following the manufacturer’s instructions and was measured in an ELISA reader at 450 nm (Biolatin CPD Reader 212).

#### Serum Total and Specific IgY Determination

##### Serum Total IgY Levels

Serum IgY levels were evaluated by ELISA test, as previously described (20) but with some modifications (7).

##### Specific IgY Anti-ETEC F4 Serum Levels

Anti-ETEC F_4_ serum levels were determined using the ELISA method developed by our research group, using the same procedure as for IgY serum levels, except that initially, the plates were coated with 50 µL of a suspension of concentrated and inactivated ETEC F_4_ strain (1 × 10^9^ UFC/mL) in carbonate–bicarbonate buffer (0.05 M, pH 9.6) and incubated overnight at 4°C (7, 20).

#### IgY Biotechnology

##### Purification of Yolk Ab

A water-soluble fraction (WSF) containing IgY from egg yolk was prepared using the water dilution method (21) with minor modifications. Briefly, the intact egg yolk was physically separated from egg white and subsequently rolled on a paper towel to remove any adherent egg white. The egg vitelline membrane was punctured, and the yolk was allowed to flow into a graduated cylinder. The egg yolk was mixed gently with six volumes of cold acidified distilled water (pH 2.5 adjusted with 0.1 M HCl). After thorough mixing, the mixture was adjusted to a pH ranging from 5.0 to 5.2 and incubated at 4°C for 12 h. The WSF was obtained by centrifugation (Beckman Coulter Avanti J-25) at 12,000 × *g* and 4°C for 20 min; the supernatant was considered as the WSF.

Avian immunoglobulin (Ig) was precipitated by sodium sulfate as described previously for the purification of IgY (22). Sodium sulfate was added at a final concentration of 20% (w/v) and stirred for 20 min at room temperature. The mixture was subsequently centrifuged at 2,000 × *g* for 30 min at 4°C. Finally, the pellet was re-suspended in PBS to the initial yolk volume and dialyzed against 0.1% NaCl solution. After dialysis, IgY samples were frozen at −20°C in plastic containers and freeze-dried for 48 h (Labconco Stoppering Try Dryer lyophilizer) (7).

##### Total IgY Yolk Concentration

The total concentration of serum IgY was evaluated by ELISA test as previously described (20) but with minor modifications (7).

##### Specific IgY Determination

Anti-ETEC F_4_-IgY levels were determined using the ELISA method developed by our research group; it was similar to that for specific IgY serum levels, except that the wells of the microtiter plates were coated with 50 µL of a suspension of concentrated and formaldehyde-inactivated ETEC F_4_ strain (>1 × 10^9^ UFC/mL) in carbonate–bicarbonate buffer (0.05 M, pH 9.6) and incubated overnight at 4°C. Serial dilutions of anti-ETEC F_4_-IgY and Control IgY (1/101 to 1/1,015) in PBS-Tween with 2% non-fat milk were incubated at 37°C for 1 h in a humidity chamber. Subsequently, an ELISA test was performed as mentioned for total IgY serum levels (7).

### Statistical Analyses

Statistical analyses were performed using Infostat software (23). Data were represented as mean ± SD. *P*-values < 0.05 were considered statistically significant (*P < 0.05; **P < 0.01; ***P < 0.001). Initially, ANOVA was used, followed by Tukey’s test.

## Results

### GALT and Spleen Response in Immunized Hens

**Figure 1:**
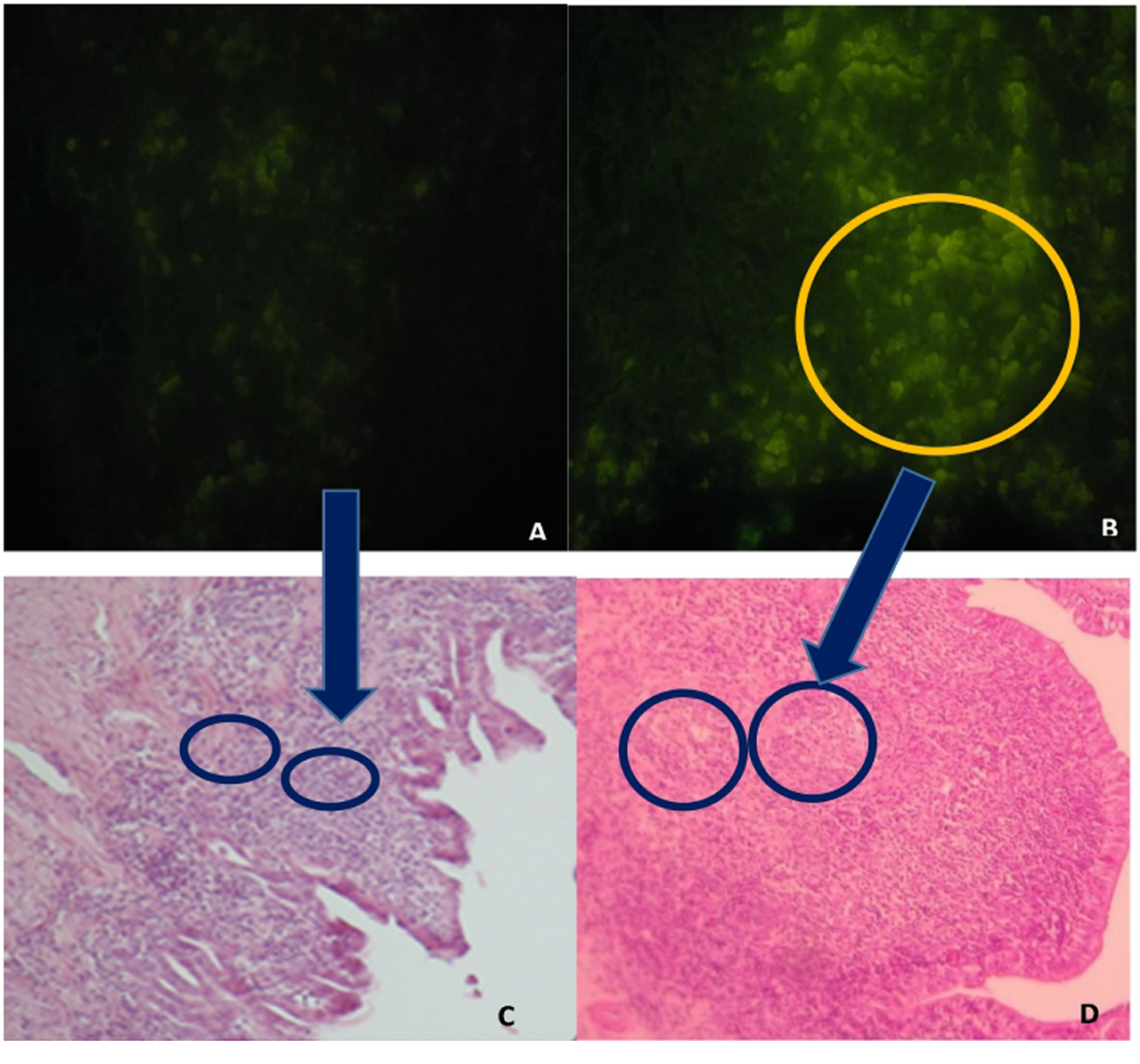
Peyer’s patches microphotography, with marked (abundant) lymphocyte production in intestinal histological sections in the F4 group and control group. **(A)** Immunofluorescence image, stained with the B lymphocyte surface antigen AV20-FitC control group. Magnification: 400×. **(B)** Immunofluorescence image, stained with the B lymphocyte surface antigen AV20-FitC. F_4_ group. Into circle highlight a completely activated germinal center. Magnification: 400× **(C)** Peyer’s patches of control group hens, stained with hematoxylin-eosin. Magnification: 100×. **(D)** Peyer’s patches of F_4_ group hens, stained with hematoxylin-eosin. Magnification: 100×.

GC in GALT and in the spleen were greater in the F_4_ group than in the Control group (Table 1). Interestingly, in GALT, the immunization triggered a greater activation in Peyer’s patches (a fivefold increase; *P* < 0.001; Figures 1B,D as compared to the Control group (Figures 1A,C)) and in esophageal tonsils (twofold increase; *P* < 0.001) in the F_4_ group (Table 1).

**Table 1:** **Germinal center area size (μm^2^) in gut-associated lymphoid tissue and in the spleen of laying hens immunized with *Escherichia coli* F_4_ bacterin**.

Group	Peyer’s patches	Esophagic tonsils	Pyloric tonsils	Cecal tonsils	Spleen
Control (*n* = 12)	78,532 ± 890***	9,786 ± 1,389***	47,316 ± 144*	4,731,651 ± 6,789*	499,318.8 ± 26,340***
F_4_ (*n* = 18)	354,899 ± 1,245***	18,897 ± 2,567***	51,027 ± 1,877*	5,102,797 ± 8,976*	5,568,072.6 ± 11,380***

The comparison was between groups (F4 and Control) into the same organ; *P < 0.05 and ***P < 0.001.

*Means that the difference between groups was significant. ***Means that the difference between groups was extremely significant.

In addition, immunization triggered cecal and pyloric tonsil activation (increased 10-fold; *P* < 0.05) in the F_4_ group as compared to the Control group. Furthermore, immunization triggered a greater activation in the spleen GC (110%; *P* < 0.001) in the F_4_ group (Table 1).

**Figure 2:**
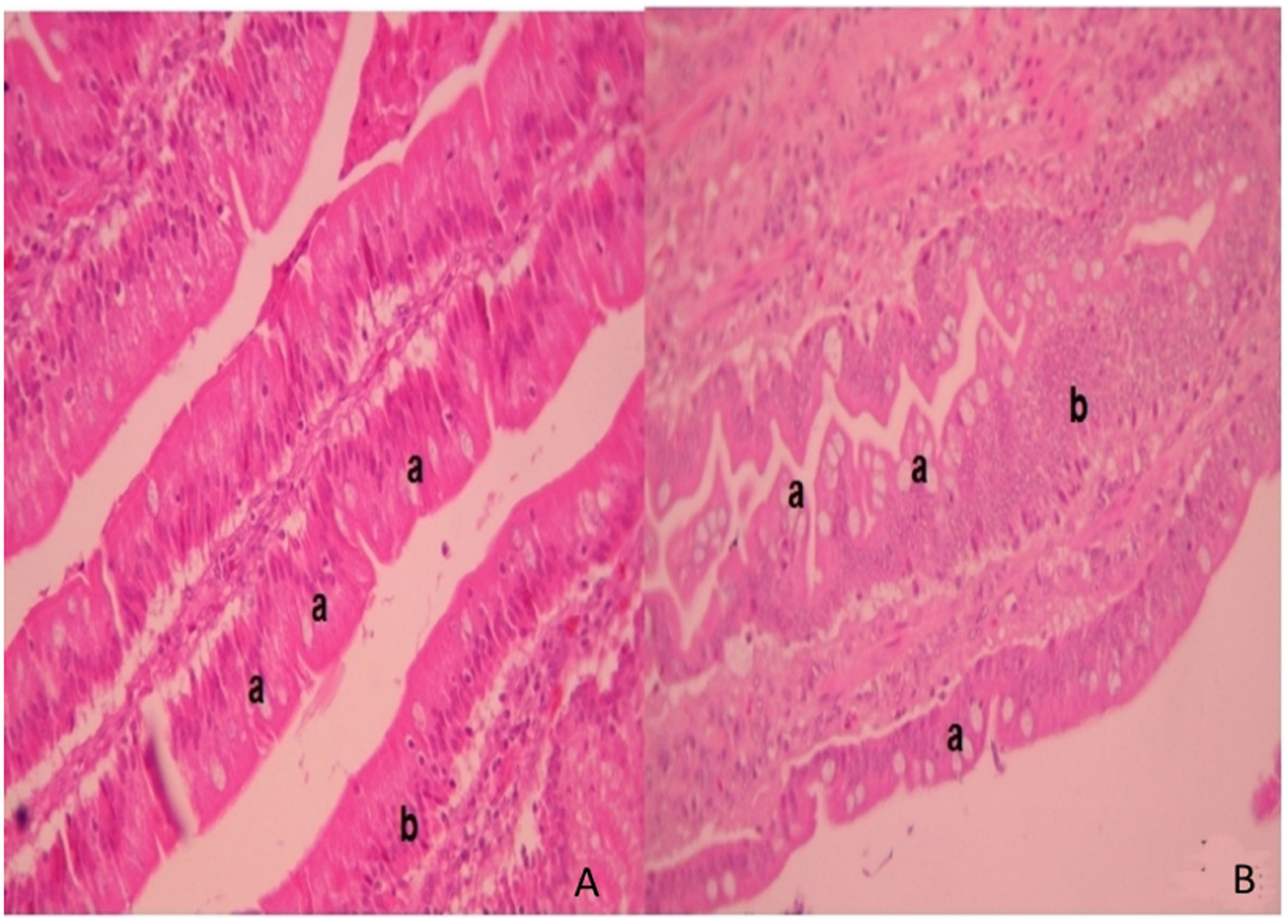
**Intestinal villi**. Intestinal histopathology microphotography in the control group (**A**) and F_4_ group (**B**) of laying hens. Into F_4_ group the villi with abundant goblet cells are highlighted (a) next to the mucus layer with an increased number of lymphocytes inside the villi (b) stained with hematoxylin/eosin. Magnification: 400×.

In ileum tissue sections, increased villi height (*P* < 0.05) and their dependent variables (area and perimeter of villi) were found in the F_4_ group. In contrast to this, in the duodenum, the variable data were similar between the F_4_ and Control groups (Table 2). Interestingly, on the villi, an increase in goblet cell numbers and the mucus layer was found. Furthermore, numerous lymphocytes (Figure 2) and plasmatic cells (Ig A-producing cells) located near to epithelial cells were found in the apical border villi in the F_4_ group (not shown). As expected, an increment in lymphocyte number in the *lamina propria* was detected in the F_4_ group.

**Table 2:** **Histomorphometric variables in the duodenum and ileum in laying hens immunized with *Escherichia coli* F_4_ bacterin**.

	Duodenum				Ileum		
Group	High (μm)	Area (μm^2^)	Perimeter (μm)	High (μm)	Area (μm^2^)	Perimeter (μm)
Control (*n* = 12)	628.08 ± 296.22	10,347.20 ± 270.81	674.84 ± 50.2	731.38 ± 193.45*	12,145.80 ± 1,198.70*	957.90 ± 150.00 *
F_4_ (*n* = 18)	1,257.86 ± 353.75	14,205.46 ± 365.02	901.99 ± 139.42	1,379.37 ± 245.70*	17,456.40 ± 820.50*	15,850.60 ± 135.40*

The comparison was between groups (F4 and Control) into the same organ (duodenum and ileum); *P < 0.05.

**Figure 3:**
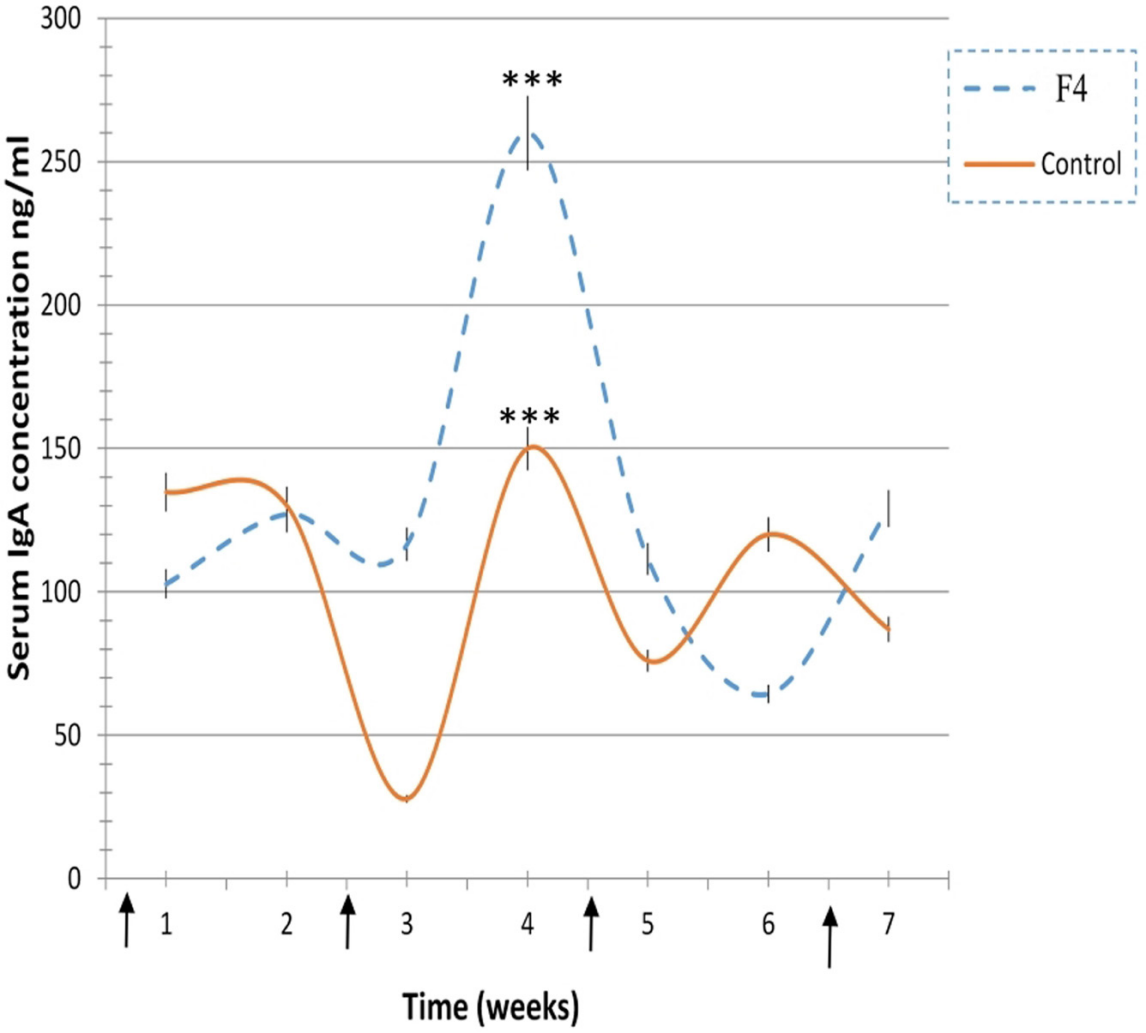
**Immunoglobulin A (IgA) concentration levels**. IgA concentration levels in laying hens from 23 to 42 weeks in the F_4_ (F_4_) and Control (C) groups. ***P < 0.001. Arrows indicate immunizations.

In concordance with the numerous plasmatic cells located near to the epithelial cells in the villi of F_4_ group samples, the levels of secreted IgA were also significantly higher (1,624 ± 131 ng/mL, *P* < 0.001) in the F_4_ than the Control group (230 ± 41 ng/mL). Furthermore, average serum IgA concentration was significantly higher (*P* < 0.05) in the F_4_ group than the Control group throughout the experimental period. High serum IgA concentration (*P* < 0.001) was reached 4 weeks after the first immunization (Figure 3).

**Figure 4:**
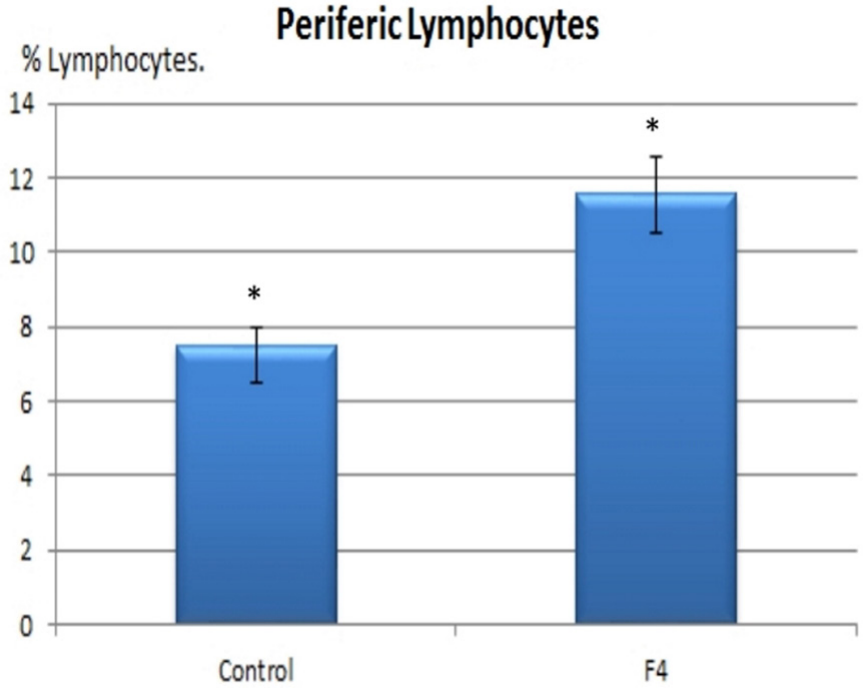
**B lymphocyte number in blood**. B lymphocyte percentages in laying hens in Control and F_4_ groups at the end of the assay (43 weeks old). *P < 0.05.

**Figure 5:**
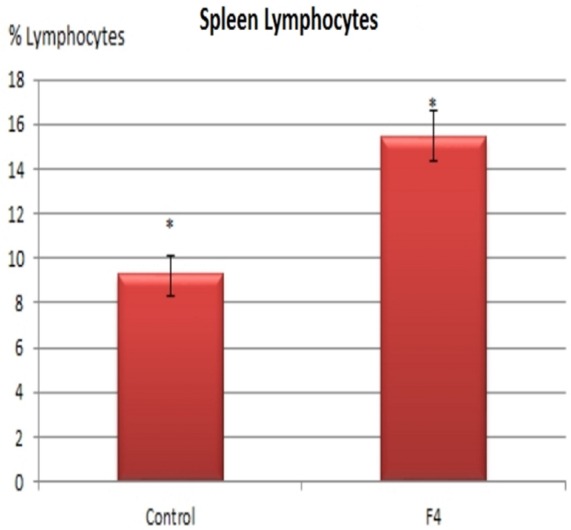
**B lymphocyte number in the spleen**. B lymphocyte percentages in laying hens in Control and F_4_ groups at the end of the assay (43 weeks old). *P < 0.05.

In concordance with the expanded GC found in GALT and in the spleen, higher percentages of B lymphocytes were found in the F_4_ group in the blood (11.8 vs. 7.8%; Figure 4) and in the spleen (15.75 vs. 9.4%; Figure 5) (*P* < 0.05).

Moreover, histological observations on the spleen corresponded well with the flow cytometry results.

### Ig Production

**Figure 6:**
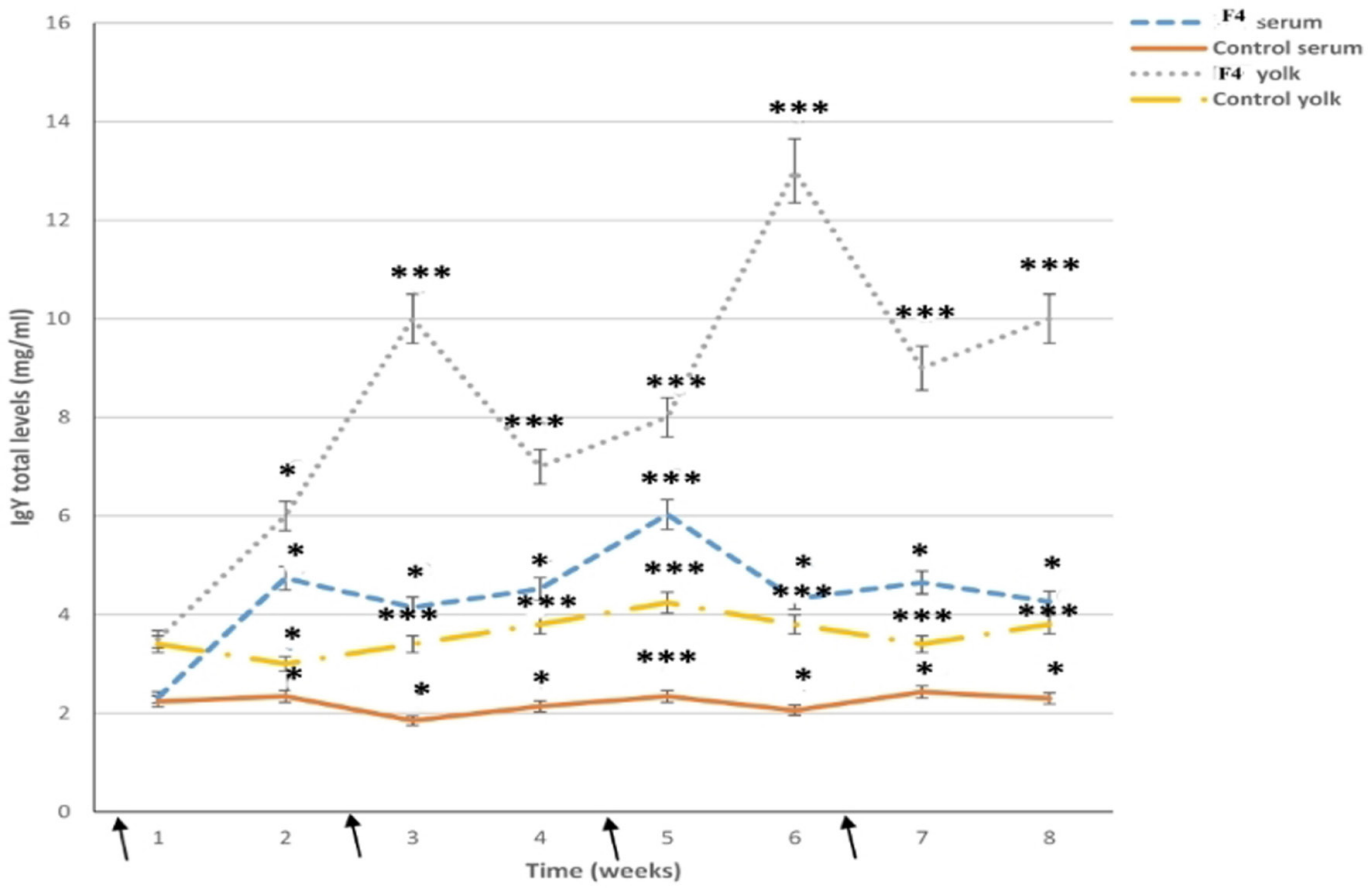
**Total immunoglobulin levels**. Serum and yolk levels of total immunoglobulin Y (IgY) in both the F_4_ and Control groups from 23 to 42 weeks *P < 0.05, ***P < 0.001. Arrows indicate immunizations

In the F_4_ group, total IgY serum levels were high for all assays (*P* < 0.001); high total IgY serum levels were reached at 2 weeks (*P* < 0.05), 5 weeks (*P* < 0.001), and 7 weeks (*P* < 0.05) after the first immunization, while IgY levels in the Control groups were kept to a minimum (Figure 6).

In the yolk, total IgY serum levels were already high after the first immunization in the F_4_ group, reached a peak by 3 weeks (*P* < 0.001; a week after the first serum peak), and they had clearly increased by 6 weeks (*P* < 0.001; a week after the second serum peak), from where they increased until the end of the research (Figure 6).

**Figure 7:**
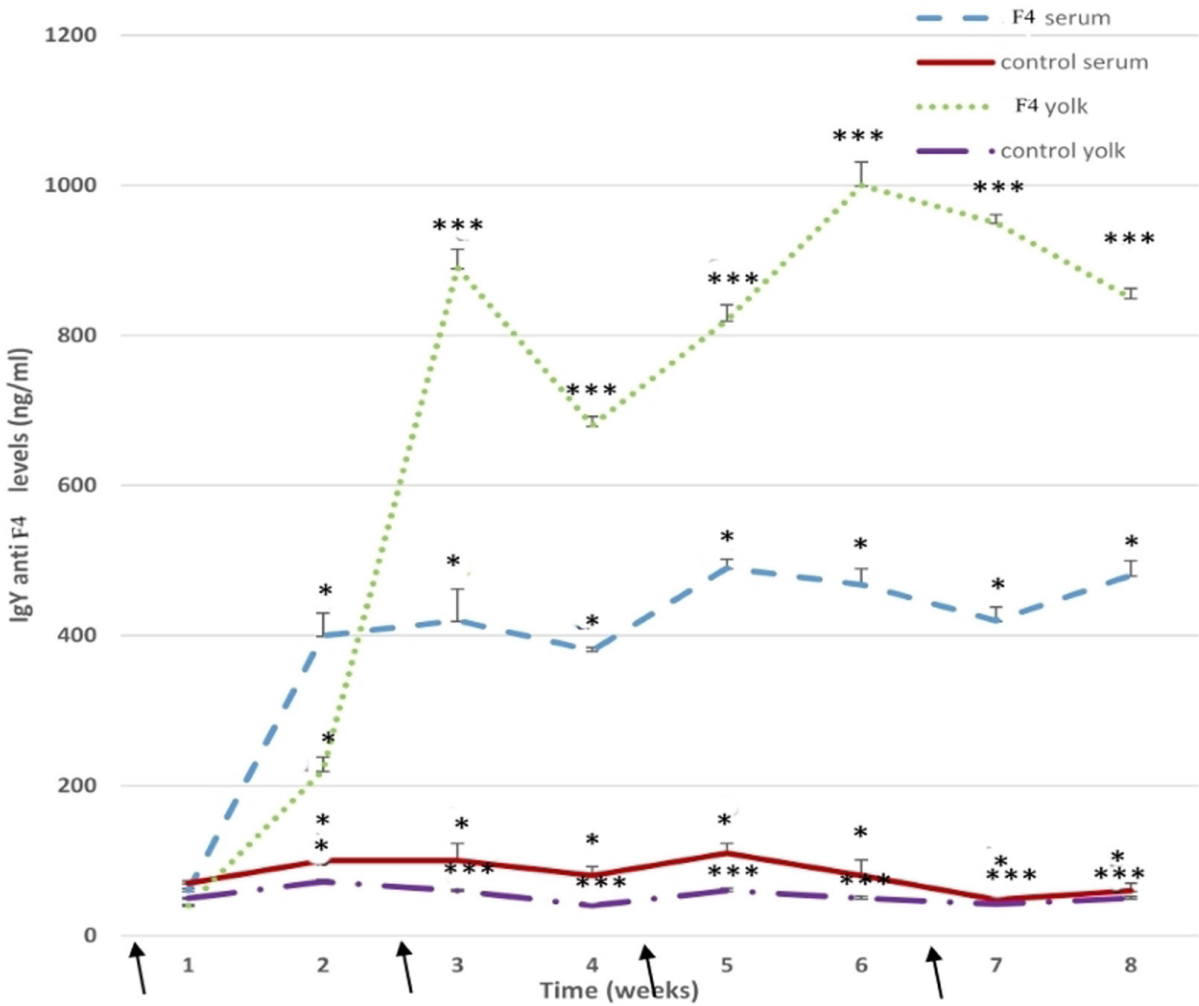
**Anti-*Escherichia coli* F4-immunoglobulin levels**. Serum and yolk levels of anti-*E. coli* F_4_-IgY in the F_4_ and Control groups from 23 to 42 weeks *P < 0.05, ***P < 0.001. Arrows indicate immunizations.

Anti *E. coli* F_4_-IgY levels in the serum remained high throughout the assay (*P* < 0.001). Some specific IgY levels increased significantly after the first immunization in the F_4_ group, high total anti-*E. coli* F_4_-IgY levels were reached at 3 weeks (*P* < 0.05), 5 weeks (*P* < 0.001), and 8 weeks (*P* < 0.05) after the first immunization (Figure 7).

In the yolk, anti-*E. coli* F_4_-IgY levels were hugely (*P* < 0.001) in the F_4_ group throughout the assay. Furthermore, high anti-*E. coli* F_4_-IgY levels were reached at 3 weeks (*P* < 0.001) and 6 weeks (*P* < 0.001) after the first immunization. This second peak was the maximum level registered in the yolk (Figure 7).

## Discussion

This work showed that intramuscular immunization of laying hens with *E. coli* F_4_ bacterin triggered a greater activation in Peyer’s patches and in esophageal tonsils than in cecal and pyloric tonsils in the GALT. Contrary to these findings, other research on avian GALT concludes that, in general, cecal tonsils are the most immunological active organ and later Peyer’s patches before immunizations with different Ags (24). The question remains as to which is the main organ being activated? It depends on the immunization schedule, the variations in the route and the system of immunogen being delivered, the inclusion of adjuvants, and the Ag presentation (whole or lysate, active, or disabled) in the vaccine, among others (2, 25–28). Also, Ag administrated orally activated mainly Peyer’s patches, followed by the cecal tonsils in GALT of mammals (25). A similar result might not be unexpected in birds as it has been generally reported that a similar mechanism of immune humoral activation takes place (29, 30). It is already known that, before the second and third immunizations, in the Peyer’s patches, bacterial fragments are picked up by the local Ag-presenting B lymphocytes as well as macrophages and dendritic cells (DCs) that activate the T lymphocytes. The activated T lymphocytes or the DCs migrate to the GC, where they activate B lymphocytes and induce the production of Ig-plasmatic cells and memory B lymphocytes (10, 22, 25, 31).

Despite the importance of other organs in the GALT, such as esophageal and pyloric tonsils, they were not studied in other researches. Interestingly, we found that both types of tonsils have been activated by the *E. coli* F_4_ bacterin. Their function on immune activation remains unclear; however, they could be related to induction and migration of B lymphocytes from Peyer’s patches to another GC in the GALT (such as cecal tonsils) by *lamina propria* and/or to direct the passage of Ags to the blood, improving the humoral IR. If the latter happens, esophagic and pyloric tonsils could potentially replace the mesenteric lymph nodes that are absent in birds but present in mammals. Perhaps, esophagic and pyloric tonsils could form a secondary barrier against Ag dissemination out of the GALT, thus avoiding some infections (29).

The intestine is the major site of development, residence, and a route of entrance for pathogenic microorganisms into the body. Any perturbation of intestinal physiology can result in substantial clinical consequences (32, 33). Hence, an effective IR in the intestine is essential. In the current research, some B lymphocytes activated in the GALT GC of the F_4_ group, migrated through the blood to the *lamina propria* inside the ileal villi, as shown in the histopathology slides. Inside the villi, several concurrent mechanisms may induce the production of Ig-plasmatic cells and memory B lymphocytes, together with T lymphocyte activation, and different soluble and/or surface molecules might be taking part. Furthermore, we detected greater ileal villi size in the F_4_ group. This is closely related to tissue turnover in the crypt region and could be induced, before bacterin immunization, through different bioactive molecules after the interaction between epithelial cells, microbiota, and immunological cells, to conserve mucosal homeostasis (11, 13, 14, 34, 35). Moreover, this increased number of lymphocytes inside the *lamina propria* of the ileum villi in the F_4_ group was consistent with the increased number of plasmatic cells registered between epithelial cells near the top of the villi (data not shown). This indicates that activated B lymphocytes in the F_4_ group have induced the production of secretory IgA in the ileal mucus layer. This is consistent with the increased IgA production in both the mucus and the serum that was recorded in this research. In the mucus, the IgA levels increased eightfold in the F_4_ group and doubled in the serum. This was expected, because IgA is the most important Ig in mucosal tissue, being the first line of immunologic defense against enteric pathogens (11, 13, 34, 36, 37). Also, IgA regulates the ecological balance of microbiota and has a fundamental role in mucosal homeostasis (37, 38). However, in the serum, IgY is the main Ig, and IgA is produced as a second option (11, 22).

Contrary to our data, in other research, no IgA serum was detected by ELISA test, after immunization of hens with an *E. coli* F_4_ bacterin (2 × 10^10^ UFC) (5). Perhaps, this difference could be related to the immunization schedule. The latter researchers used a low dose of bacteria combined with Freud’s adjuvant and Montanide ISA 25 adjuvant, which induced a low IR that could not be detected by ELISA assay. In the current research, we used a higher concentration of *E. coli* F_4_ (1 × 10^9^ UFC) with aluminum hydroxide as the adjuvant, so we obtained a high mean IgA levels after immunization.

According to the increased mucosal IgA levels registered into the bacterin-immunized hens, we detected an increment in intestinal protection, produced by an increase in both goblet cell number and their mucus production.

This work revealed that activated B lymphocytes entered the general circulation and caused a 50% increase in the number of circulating B lymphocytes, as recorded in the flow cytometry study. This is the response of the activation in GALT and the spleen, as we registered in the histopathologic and immunofluorescence study and in flow cytometry in the spleen. These data are consistent with GC activation in both the GALT and the spleen (22, 25). Following *E. coli* F_4_ immunization, the efflux of B lymphocytes and specific Ig from the GALT and the spleen increased, and both were delivered into the periphery in order to identify and remove bacterial Ags (22, 25). In other research in 24-week-old broilers, an important increase was found in both B and T lymphocyte number, post intravenous immunization with *E. coli* bacterin (1 × 10^10^ UFC/mL), at 0, 3, 5, and 7 days and then each 7 days until 21 days (39). It will be necessary in the future, to measure T lymphocyte populations in laying hens intramuscularly immunized with *E. coli* F_4_ bacterin, for a better understanding of the induction of humoral IR.

The spleen is the second most important secondary lymphatic organ after the MIS, controlling the blood and generating an IR to Ags through specific Ig secretion and clonal expansion of B lymphocytes in the GC (40). After *E. coli* immunization in the F_4_ group, B-lymphocyte number, as determined by flow cytometry, increased by about 40% inside the activated GC, similar to that registered in the immunofluorescent study mentioned earlier. Contrary to our data, in 8-week-old Leghorn males, immunized with *E. coli* (serotype 055: B5; 8 mg/kg, intravenously), no change was registered in B lymphocyte number from blood or the spleen at 24 h post immunization. The authors concluded that there was a redistribution of B lymphocytes into the spleen and into other secondary lymphatic organs (41), but the real reason was that these researchers did not consider the time necessary to induce clonal expansion in B lymphocytes inside the GC, B lymphocyte activation, and posterior B lymphocyte migration into the blood (>36 h required) (31).

With reference to IgY production, B lymphocyte activation in the spleen GC of *E. coli* F_4_ immunized hens was reflected by the 100% increment in specific serum IgY. This serum IgY is then transported to the yolk, where IgY levels are concentrated (2, 42). According to the latter authors, the yolk registered an increment of 600% in specific IgY in bacterin-immunized hens. Interestingly, the first peak in specific serum IgY was noticed after the second immunization, and a week later the first peak in yolk IgY was registered.

Immunoglobulin Y levels in our research were double the levels found in other research, where hens were immunized with *E. coli* (6, 9). Perhaps, the difference might be due to the Ag (different strain or low concentration) and the adjuvant employed [Adjuvant Freud Complete (AFC) or Adjuvant Freud Incomplete (AFI) or AFC-AFI combination] that was different to the aluminum hydroxide used in the current study. It is well known that AFC produces serious damage at the site of injection, affects the health of hens, and also affects egg production (43). One notable advantage of using IgY biotechnology is animal welfare, but if AFC is used this benefit can be disregarded (44). In the current research, we used aluminum hydroxide, which generates a good IR that is focused in humoral IR and in cellular IR in lower amounts. Furthermore, *E. coli* was inactivated through the use of formaldehyde in the current study to preserve the vita-PAMPs (Molecular Pattern Associated to Pathogens related to vitality), which contribute to high IRs, both innate and adaptive (39).

In previous research, specific IgY levels in hens immunized with *E. coli* 0157:H7 and AFI were greater than in our data (20). This discrepancy from our work might be due to the strain used; 0157:H7 is an enterohemorrhagic strain, which presents great virulence factors and is capable of producing a verotoxin (Shiga toxin), which is responsible for generating high morbidity and mortality levels in humans; it is related to uremic–hemolytic syndrome (45). Furthermore, they used whole bacteria; so all virulence factors were contained within the vaccine. In our research, we used *E. coli* F4 lysate, a strain with lower pathogenicity for birds because it is a strain owned from pigs (4).

## Conclusion

Laying hens, intramuscularly immunized with *E. coli* F_4_, activated both the mucosal and the systemic immune system. Mucosal protection is produced through the activation of the GC, essentially in the Peyer’s patches and in the esophagic, pyloric, and cecal tonsils, then later in the GALT. This activation produces clonal expansion of B lymphocytes, which migrate through the *lamina propria* to the intestinal villi. There, the bacterin produces APC and T lymphocyte activation, which activates B lymphocytes to produce high levels of IgA, both mucosal and systemic. Furthermore, after interaction between immune and absorptive cells and microbiota, ileal villi protection is increased through mucosal hypersecretion produced by the increased number of goblet cells.

Subsequently, a portion of B lymphocytes, after their clonal expansion, migrated from the blood to the spleen, where they activated other B lymphocytes in the spleen GC, resulting in systemic protection by high specific IgY serum levels. After one week, this IgY was present in the egg yolk.

These results suggest that GALT is a key immunologic tissue inside the MIS, working as a “command center” of humoral reaction.

## Ethics Statement

This study received formal approval from the ethics committee of the Universidad Nacional de Río Cuarto. In order to guarantee a safe, correct and careful use and handling of experimental animals, the investigators proceeded according to the ethical guidelines of animal welfare committee (59) (see Materials and Methods).

## Author Contributions

Conceived and designed the experiments: MP, FA, AN, RM, AV. Performed the experiments: MP, AM, AN, FA, AV. Analyzed the data: MP, AV. Wrote the paper: MP, AV, AM, FA.

## Conflict of Interest Statement

The author declares that the research was conducted in the absence of any commercial or financial relationships that could be construed as a potential conflict of interest. The reviewer, MS, and handling editor declared their shared affiliation, and the handling editor states that the process nevertheless met the standards of a fair and objective review.

## Abbreviations

Ab, antibody; Ag, antigen; BALT, bronchial-associated lymphoid tissue; CMF, tamponed Saline solution without Ca and Mg; CFU, colony forming unity; DCs, dendritic cells; ELISA, enzyme-linked immunosorbent assay; *E. coli, Escherichia coli*; FITC, fluorescein isothiocyanate; GALT, gut-associated lymphoid tissue; GC, germinal center; H/E, hematoxylin-eosin; IR, immune response; Ig, immunoglobulin; IgA, immunoglobulin A; Ig G, immunoglobulin G; IgY, immunoglobulin Y; MALT, mucosae-associated lymphoid tissue; MIS, mucosal immune system; NALT, nasopharyngeal lymphoid tissue; PBS, phosphate buffered saline; RPMI, Roswell Park memory institute medium; TMB, 3, 30, 5, 50-tetramethylbenzidine; WSF, water-soluble fraction.
